# COUNTERPOINT: Liquid Markers for Risk Stratification of Pulmonary Nodules, Ready for Prime Time? Not Yet!

**DOI:** 10.1016/j.chpulm.2024.100070

**Published:** 2024-06-11

**Authors:** Tejaswi R. Nadig, Robert Smyth, Brett C. Bade

**Affiliations:** a Section of Interventional Pulmonology, Department of Medicine, Memorial Sloan Kettering Cancer Center, New York City, NY; b Section of Pulmonary, Critical Care, and Occupational Medicine, Department of Internal Medicine, University of Iowa, Iowa City, IA; c Section of Pulmonary, Critical Care, and Sleep Medicine, Department of Medicine, Northwell Health, New York City, NY

Lung nodules present a diagnostic challenge. As pulmonologists, our tasks are the following: (1) to distinguish malignant from benign nodules, (2) to facilitate early diagnosis for malignant nodules, and (3) to minimize invasive procedures in those with benign nodules. Because most pulmonary nodules are benign, our role is to find the needle in an inexorably growing haystack. Even before lung cancer screening was recommended, the widespread use of CT scan resulted in a rising incidence of incidental pulmonary nodules in the United States, with estimates of 1.5 million per year.^1^^,^^2^ As lung cancer screening rates continue to increase, the burden of lung nodule management will certainly grow.

Lung nodule management relies on a pulmonary nodule’s pretest probability of malignancy (pCA) using patient factors and imaging to inform clinical decisions.^3^ Because biomarkers have the potential to noninvasively influence a nodule’s pCA, the availability of a reliable and effective biomarker would immediately change practice patterns. Specifically, an effective biomarker would allow physicians to pursue procedures in patients at the highest risk of malignancy and avoid unnecessary procedures and costs in patients at lower risk. Although such biomarkers hold exciting potential, we argue that the currently available biomarkers are not sufficiently accurate or reliable and therefore are not ready for prime time. Here, we briefly review the state of biomarker testing, recent literature, and our own assessment of biomarkers’ current clinical utility. We will not address already validated biomarkers used in the diagnosis or categorization of lung cancer (eg, Programmed death-ligand 1 (PD-L1), targetable mutations).

## State of the Science

**Figure 1:**
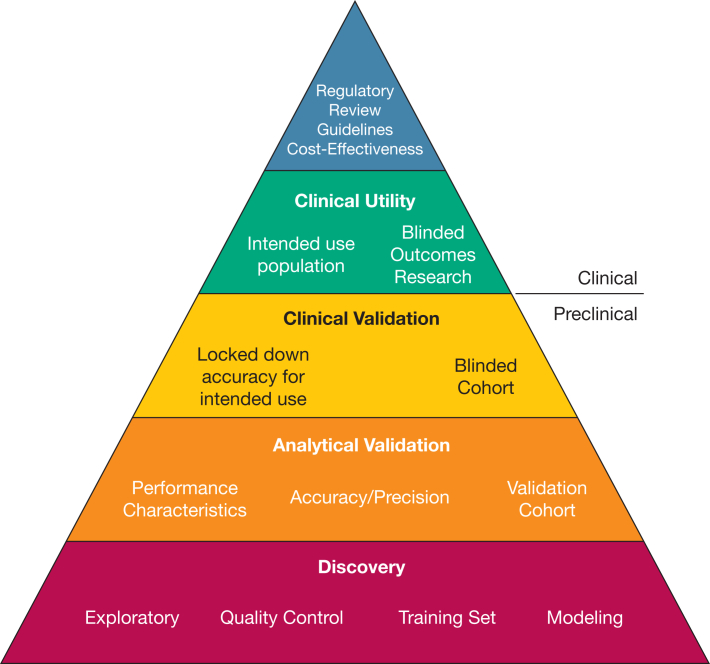
Stages of biomarker development. Reprinted with permission from Sears et al^5^

Biomarkers must go through several phases before readiness for clinical use (Fig 1). The 2017 policy statement of the American Thoracic Society (ATS)^4^ on molecular biomarkers for lung cancer outlined this process (Table 1). The ATS statement outlines how studies should be designed and reported, include definitions of minimal accuracy, and include scenarios where patients are most likely to benefit.^4^ The ATS statement categorizes biomarkers into risk prediction, cancer detection, and diagnosis. This discussion will focus on cancer detection.

**Table 1:** Key Points From American Thoracic Society Policy Statement

Aspect	Key Considerations
Biomarker validation	• Rigorous validation is crucial before clinical use. • Must demonstrate high sensitivity and specificity. • Validation should involve large, diverse patient cohorts.
Clinical utility	• Biomarker should improve early detection over existing methods. • It should provide actionable information for physicians. • Consider cost-effectiveness and ease of implementation.
Biomarker development	• Early research and discovery stage precede validation. • Multiple potential biomarkers may need to be explored. • Focus on noninvasive or minimally invasive methods.
Regulatory approval	• Compliance with regulatory agencies (eg, FDA) is critical. • Requires robust clinical trial data and safety assessment. • Biomarkers may receive different regulatory classifications.
Biomarker performance	• Regular monitoring of performance in real-world settings. • Updates and refinements based on ongoing research. • Consider long-term follow-up for patient outcomes.
Ethical and legal	• Adherence to ethical guidelines and patient consent.
Considerations	• Address legal and privacy issues related to data sharing. • Ensure equitable access to biomarker-based tests.
Cost-benefit analysis	• Assess the overall cost-effectiveness of biomarker testing. • Weigh benefits against potential harms and costs.

FDA = US Food and Drug Administration.

As previously highlighted, incorporation into clinical practice extends beyond readiness for clinical use. More specifically, a useful biomarker must improve prediction of a nodule’s pCA, influence clinical decision-making, reduce unnecessary procedures, and lower lung cancer mortality. Given the potential for harm from inaccurate testing (eg, delayed diagnosis of malignancy), several challenges and limitations must be addressed before a biomarker is adopted.

## Background: Biomarkers Improve Cancer Risk Prediction…a Little

**Figure 2:**
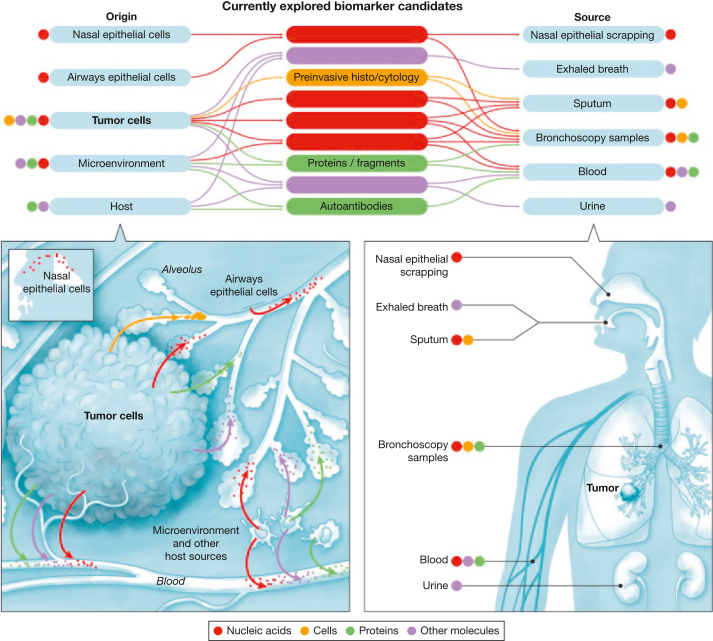
Lung nodule biomarker sources. Reprinted with permission from Seijo et al^6^

Lung nodule biomarkers may include DNA, RNA, and proteins derived from many tissue sources (Fig 2). The current literature may be broadly categorized into biomarkers aimed to optimize patient selection for lung cancer screening and those distinguishing benign from malignant pulmonary nodules. This organization serves as a good framework for our discussion. Despite extensive research, few biomarkers have emerged from the initial discovery phase and progressed to clinical validation/utility studies. There is additional uncertainty regarding which biomarkers should be used (and when). Here we discuss some of the most promising candidates and the lessons from the literature.

Airway Epithelial Gene Expression in the Diagnosis of Lung Cancer (AEGIS) was one of the first biomarker studies in lung nodules. AEGIS showed that including a bronchial airway gene expression classifier (from a bronchial brushing) could improve the diagnostic performance of traditional bronchoscopy in people with active or previous tobacco use with suspected lung cancer.^7^ In 639 patients, the combination of the classifier plus bronchoscopy had a sensitivity of 96% in AEGIS 1 and 98% in AEGIS 2 (compared with 74% and 76% for traditional bronchoscopy, respectively). Although the results were encouraging, bronchial genomic classifier has not gained widespread clinical use.

The Pulmonary Nodule Plasma Proteomic Classifier (PANOPTIC) combines serum markers with clinical factors and is one of the few serum biomarkers to reach clinical use. The defining publication was a prospective, multicenter observational study assessing the accuracy of a serum derived protein-based blood test in identifying benign nodules in patients with a pCA ≤ 50%.^8^ Thus, the classifier is intended to reduce the number of invasive procedures performed on patients with benign disease. When integrated with clinical risk prediction models, it had a sensitivity rate of 97% (95% CI, 82%-100%), a specificity rate of 44% (95% CI, 36%-52%), and a negative predictive value of 98% (95% CI, 92%-100%) in distinguishing benign from malignant nodules. The analysis suggested a 40% reduction in invasive procedures.^8^

Preliminary studies suggest that tumor-derived DNA may identify early and advanced stage lung cancers.^9^^,^^10^ In cell-free DNA, Lung EpiCheck (Nucleix) detects lung cancer-associated hypermethylation. The two validation cohorts in the preliminary study achieved areas under the curve of 0.882 and 0.899.^9^ Leal et al^10^ investigated cell-free DNA fragmentation patterns (DNA evaluation of fragments for early interception score) in a prospective study of 296 individuals with symptoms of lung cancer. Median DNA evaluation of fragments for early interception scores were higher for those with lung cancer (n = 98) than those without cancer (n = 198; 0.94 vs 0.19; *P* < .001).^10^

Early CDT Lung (OncImmune) aims to detect the presence of antibodies to lung cancer-associated antigens in the peripheral blood. A systematic review reported the diagnostic accuracy of this test in 695 patients with pulmonary nodules.^11^ This analysis reported a sensitivity of approximately 20.2% and specificity of 92%. There were no data on its clinical utility beyond its diagnostic accuracy. Because combined impact of Early CDT and existing clinical models (ie, Brock, Herder) is not available, it is unclear how this test would better inform our current practice.

To summarize, multiple available studies show that biomarkers have the potential to improve risk pCA estimation in patients with lung nodules. Thus, the background literature is cause for cautious optimism that our evaluation of lung nodules will greatly improve in the future. However, it is similarly clear that further study is needed before these markers can be considered ready for widespread clinical use.

## Conclusion: Biomarkers for Lung Nodule Management Are Not Ready for Prime Time

Current and emerging evidence hints at a future where lung nodule assessment and lung cancer screening decision-making will include biomarkers. However, three aspects of currently available data emphasize that biomarkers for lung nodule evaluation are not ready for widespread clinical use. First, the sensitivity and specificity of these biomarkers must improve to reliably differentiate benign from malignant nodules. Inaccurate results can lead to unnecessary anxiety, unnecessary interventions in patients, or missed opportunities for early intervention. Moreover, large-scale clinical trials are needed to assess their clinical utility, cost-effectiveness, and impact on patient outcomes. Second, physician education on the appropriate use and interpretation of these biomarker results is currently lacking. Tanner et al^12^ investigated how a rule-in blood test would change physicians' management of pulmonary nodules. Despite the use of a rule-in biomarker test, physicians often misinterpreted the results and failed to follow preexisting American College of Chest Physicians guidelines.^12^ Therefore, significant education is required prior to the widespread implementation of these tests. Finally, societal statements and lung nodule management guidelines have yet to incorporate biomarker testing into risk assessment or management algorithms.^3^^,^^13^ In combination, these findings should give us pause. In conclusion, pulmonologists are anxiously awaiting better tools to distinguish benign from malignant nodules. Although it is tempting to broadly implement promising products, the lung cancer community should insist on additional evidence of clinical utility before changing practice. Rather than prime time, it seems clear that our current biomarker programming remains closer to early morning or late-night television. We now return you to your regularly scheduled programming.

## Funding/Support

This manuscript did not receive dedicated funding.

## Financial/Nonfinancial Disclosures

The authors have reported to *CHEST Pulmonary* the following: B. C. B. reports relationships with Delfi Diagnostics, Biodesix, Inc, and Nucleix Ltd that includes site principal investigator for clinical trial, an investigator-initiated research grant from the American Cancer Society and investigator-initiated research support from the VA Central Office. None declared (T. R. N. and R. S.).
